# Effects of sterol regulatory element-binding protein (SREBP) in chickens

**DOI:** 10.1186/1476-511X-11-20

**Published:** 2012-02-06

**Authors:** Fahimeh Alipour, Ahmad Hassanabadi

**Affiliations:** 1 Department of Animal Science, Faculty of Agriculture, Ferdowsi University of Mashhad, Mashhad 91775-1163, Iran

**Keywords:** Sterol regulatory, element binding protein, expression, chickens

## Abstract

Sterol regulatory element binding protein- 1 and -2 (SREBP-1 and -2) are key transcription factors involved in the biosynthesis of cholesterol and fatty acids. The SREBP have mostly been studied in rodents in which lipogenesis is regulated in both liver and adipose tissue. There is, though, a paucity of information on birds, in which lipogenesis occurs essentially in the liver as in humans. Since a prelude to the investigation of the role of SREBP in lipid metabolism regulation in chicken, we review Size and Tissue expression Pattern of SREBP and role of this protein in chickens.

## Background

Sterol regulatory element binding proteins (SREBP) are basic helix-loop-helix-leucine zipper (bHLH-Zip) transcription factors that play an important role in controlling genes involved in the biosynthesis of cholesterol and fatty acids [1]. The SREBP precursors of about 1150 amino acids in length need cleavage by a sterol-dependent proteolytic process [2]. The N-terminal part (about 450 amino acids, having the bHLH-Zip motif and the transcription activating domain) is then released into the nucleus, where the transcriptional activation of the target genes occurs. SREBPs are synthesized while inactive precursor proteins that are embedded in endoplasmic reticulum membranes [3,4]. To become transcriptionally active, precursor SREBP is escorted to the Golgi apparatus, where it undergoes a sequential 2-step proteolytic cleavage catalyzed by site-1 protease and site-2 protease [5]. Therefore, two SREBPs, designated SREBP-1 and -2, have been isolated and cloned from several mammalian species [6,7]. The SREBP-1 gene generates two isoforms SREBP-1a and -1c, by another transcription start sites [8].

This procedure releases an amino-terminal SREBP fragment that is referred to as the mature form. Mature SREBP is transported into the nucleus, wherever it binds sterol regulatory elements (SRE) of genes involved in biosynthesis of lipid. Three isoforms of SREBP have been identified in mammals. Two of these isoforms, designated SREBP-1a and SREBP-1c, are expressed from the same gene. They vary in sequence at their amino termini by reason of utilize of alternative promoters and leading exons. The third isoform, designated SREBP-2, is expressed from a separate gene. SREBP-1c and SREBP-2 are the major isoforms of SREBP expressed in mammalian liver [5]. Several studies recommend that the SREBP-1 isoforms are more selective in activating fatty acid biosynthesis genes, while SREBP-2 is more specific for controlling cholesterol biosynthesis. These researches include on hepatic lipogenic gene expression in genetically modified mice characterized by over expression or disruption of SREBP [9-12] in addition to studies on physiological changes of SREBP levels in normal mice after treatment by insulin or after dietary manipulation for instance placement on high carbohydrate diets, unsaturated fatty acid-enriched diets or fasting-refeeding regimens [11-18]. As a result, SREBPs coordinate the synthesis of the two major building blocks of membranes, fatty acids, and cholesterol.

### Size and tissue expression pattern of chicken srebp mRNA

**Figure 1:**
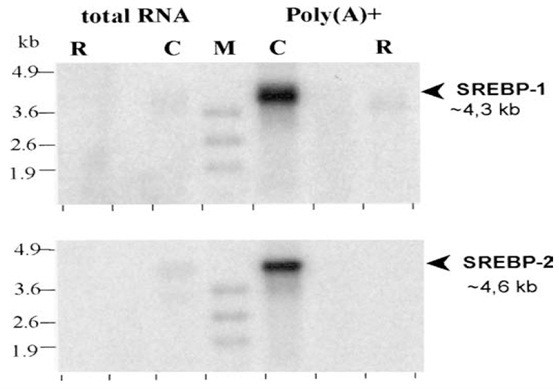
**Regulation of G_i2, LDLR, and G_s promoter activities by growth of embryonic chick atrial cells in LPDS (A) and by overexpression of SREBP in cells cultured in FCS (B)**. Effect of increasing concentrations of SREBP on G_i2 promoter activity in cells cultured in FCS (C).

Assaf et al. (2003) determine the sizes of mRNA encoded by the chicken SREBP-1 and SREBP-2 genes, by Northern blot analysis with 20 μg of total RNA or 5 μg of poly (A)^+ ^RNA prepared from chicken liver (Figure 1). For each gene, single transcripts of approximately 4.3 kb for SREBP-1 and 4.6 kb for SREBP-2 were clearly visible with chicken liver poly (A)^+ ^RNA, whereas the hybridization signal obtained with total RNA was undetectable. In this experiment no crosshybridization was observed between chicken probes and rat RNA [19].

The pattern of a single band for SREBP-1 is alike to that reported previously in chicken [20]. The sizes of chicken SREBP-1 and SREBP-2 mRNA are relatively similar to those reported for rat or human: ~ 4 kb for SREBP-1 [6,7] and ~5 kb for SREBP-2 [21]. SREBP-1 and SREBP-2 were expressed in a broad variety of tissues in chicken. The SREBP-1 was expressed preferentially in the liver and uropygial gland, the latter expressing three times further SREBP-1 than the former. The expression in other tissues examined (adipose tissue, heart, lung, kidney, intestine, muscle, brain, and testis) was approximately two to five times lower than that in the liver, while the spleen tissue presented a very low relative mRNA level. The SREBP-2 expression was greatly variable between the two birds analyzed. Most of the tissues analyzed expressed SREBP-2 mRNA approximately equal to the liver, except for skeletal and cardiac muscles and spleen, in which expression level of SREPB-2 was roughly less than half of that in the liver of whatever bird was investigated. Chicken hepatocytes and hepatoma LMH cells expressed similar levels to those observed in the liver for the two genes [19].

The tissue expression data by other research show that SREBP-1 was preferentially expressed in two organs, the uropygial gland, in which fatty acids utilized to protect the bird feathers are synthesized and stored [22,23], and the liver, which is the main site of fatty acid synthesis in chicken [23,24]. In contrast, SREBP-1 was weakly expressed in tissues where lipogenesis is very low, such as adipose tissue, muscles, intestine, and testes. Similar differences in the relative expression level of SREBP-1 between the liver and the adipose tissue have been previously reported in chicken [21]. The SREBP-2 expression seems to be roughly similar in most of the tissues examined. As a result, these studies are consistent with the important role of SREBP-1 in the regulation of lipogenesis, which has until now been mainly described in mammals [25,26,11,15,14,18,13]. This is the case for nonrodent mammals and birds, in which only one lipogenic site is strongly active: the adipose tissue in pigs [27] and the liver in birds [3]. Gondret et al., (2001) showed that in these two species, there is a close relationship between the tissue specificity of Fatty acid synthase (FAS) expression and the level of ADD-1/SREBP-1 but not SREBP-2 mRNA. They also reported that the tissue distribution of SREBP-1 mRNA between species is paralleled by commensurate variations in the nuclear concentration of SREBP-1 protein. This finding suggests a direct role for SREBP-1 in the relative level of FAS protein between tissues and species [20]. However, some studies have reported the existence of posttranslational modifications such as phosphorylation [27], which might modulate SREBP transcriptional activity.

### Role of srebp in the regulation of g_α2 _expression

**Figure 2:**
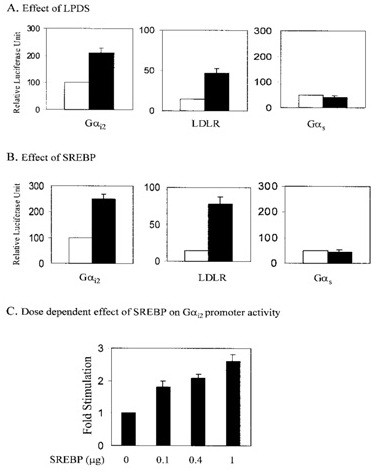
**Analysis of chicken sterol response element binding protein (SREBP)-1 and SREBP-2 RNA levels in the liver**. The SREBP probes were hybridized to 5 μg of poly(A) RNAor 20 μg of totalRNAextracted from liver of rat (R) or chicken (C) species.

The balance between the responses of the heart to parasympathetic and sympathetic stimuli find outs not only the rate and force of contraction, although may perhaps play a role in regulating cardiac excitability [28]. Parasympathetic regulation of heart rate involves the binding of acetylcholine to M2 muscarinic receptors localized primarily on atrial myocytes. Acetylcholine binding consequences in the dissociation of the heterotrimeric G protein, Gα2, into α12 and βγ subunits and activation of the inward rectifying K^+ ^channel, GIRK, with a resulting enhance in diastolic depolarization and a diminish in beat rate.2 The conclusion that the increased expression of Gα12 might influence cardiac excitability and parasympathetic response is steady with report in which the overexpression of Gα12 in the atrioventricular (AV) node of the pig via an adenoviral vector decreased AV conduction and slowed the ventricular answer to atrial fibrillation and interfered with results of β-adrenergic stimulation on cardiac excitability [29]. Haigh et al. (1988) demonstrated that growth of embryonic chick atrial cells in medium supplement by lipoproteindepleted serum (LPDS) consequence in a marked enhance in their response to muscarinic stimulation and a reciprocal decrease in their response to β -adrenergic stimulation [30]. These effects of LPDS were reversed by adding back the serum LDL part to the culture medium. results of Haigh et al. (1988) and Gadbut et al. (1997) reported that increase in the parasympathetic response in cells cultured in LPDS was associated with an enhance in the expression of muscarinic receptors, Gα12 and GIRK1[30,31]. These results proposed that a unique relationship might exist between lipid metabolism, the regulation of genes involved in the parasympathetic response of the heart and the response of the heart to parasympathetic stimulation. So as to determine whether SREBP played a role in the induction of Gα12 expression in response to growth of cells in LPDS, Park et al (2002) showed that regulation of the Gα12 promoter luciferase reporter (Gα12-Luc) in embryonic chick atrial cells cultured in LPDS and in cells cotransfected with a vector expressing SREBP-1a. Results demonstrate that in embryonic chick atrial cells transfected with Gα12 -Luc (pGL3- G α12 -2.2 kb); growth in LPDS resulted in a 2.0 ± 0.1-fold increase in Gα12 promoter Activity (Figure 2). This effect was specific for Gα12 as showed by the absence of an effect of LPDS on chick Gα12 promoter activity (Figure 2A). Cotransfection of cells cultured in media supplemented with FCS with Gα12 -Luc and pcDNASREBP-1a caused in a 2.5 ± 0.1-fold increase in Gα12promoter activity in contrasted with control (Figure 2B), while cotransfection with LDLR-Luc and pcDNA-SREBP-1a resulted in a 5.4_0.5-fold enhance in LDL receptor promoter activity. SREBP-1a had no effect on Gα12 promoter activity (Figure 2B). Moreover, stimulation of Gα12 promoter activity by SREBP-1a was concentration dependent as demonstrated by the increase in Gα12 promoter activity with increased concentration of pcDNA-SREBP-1a in the transfection medium (Figure 2C) [32].

Sheng et al (1995) reported that hamsters use both HMG-CoA reductase inhibitors and sterol depletion with bile acids have demonstrated that the expression of SREBP-2 and SREBP-1 is differentially regulated.17 Moreover, in transgenic mice expressing dominant activating forms of SREBP-2 and SREBP-1, SREBP-2 has been shown to be a comparatively selective activator of cholesterol synthesis as opposed to fatty acid synthesis,18 while SREBP-1 has been shown to be relatively specific for the regulation of enzymes involved in fatty acid biosynthesis [9,12]. Also other researchers demonstrated a role for SREBP-1 as a mediator of insulin action on the expression of glucokinase in liver. As little is known regarding the function of SREBP in the heart, these studies show a mechanism for the regulation of gene expression by SREBP in cardiac tissue. The result that SREBP-1 rather than SREBP-2 regulates Gα12promoter activity is constant with the observation that SREBP-1 is relatively specific for the regulation of genes not involved in cholesterol biosynthesis. Furthermore, this is the first demonstration of SREBP regulation of expression of a protein that does not play an obvious role in lipid metabolism, fatty acid synthesis, or the generation of precursors to fatty acids [32].

### Molecular cloning of chicken srebp

A 604 bp SREBP-2 fragment was first obtained by RTPCR using the advance hu600S primer derived from human SREBP-2 sequence (NM_004599) and corresponding to amino acids 144 to 152 of human SREBP-2, and the reverse ch600AS primer chosen from a 386 bp chicken SREBP-1 sequence (AF278697) encompassing the bHLHZip domain known to be nearly conserved between SREBP-1 and SREBP-2 genes. An 850 bp fragment was next obtained by RT-PCR by the forward ch850S primer derived from the formerly amplified 604 bp chicken SREBP-2 fragment, and the reverse human hu850AS primer corresponding to amino acids 551 to 561. The sequence of these two clones corresponded to a partial chicken SREBP-2 cDNA of 1,190 bp that was deposited in GenBank/EMBL database under the accession number AJ310769. The 1,190 bp SREBP-2 sequences was finally extended in the 5' direction, by RT-PCR using the forward hu5'UTRS primer corresponding to nucleotides 36 to 57 of the 5' UTR region of the human SREBP-2 sequence, and the chicken reverse ch5'AS primer. The RT-PCR was followed by a nested PCR by means of the human forward hu5'UTRnS primer corresponding to nucleotides 62 to 84 of the 5' UTR region and the reverse chicken ch5'nAS primer. The total 1,582 bp SREBP-2 chicken sequence obtained was deposited in GenBank/EMBL database, under the accession number AJ414379. The degrees of relationship of the 1,582 bp SREBP-2 chicken sequence with human, mouse and hamster SREBP-2 (species for which full cDNA are available) were 81 to 82% for the DNA sequences and 77 to 79% for the forecasted protein sequences [19]. Also, the different residues required for membranebound SREBP cleavage by site-1 and site-2 proteases [33,2] are conserved among human, mouse, hamster, and chicken, especially the RXXL tetrapeptide necessary for the site-1 cleavage, the tetrapeptide DRSR, which marks the end of the cytosolic N-terminal segment of SREBP and the leucine located 3 residues after the DRSR sequence, which marks the end of the sequence of the mature N-terminal fractions of SREBP, which enter into the nucleus to activate gene transcription. Consequently, the chicken SREBP-2 sequence consists of the whole sequence encoding the mature N-terminal part of this transcription factor. As observed in humans, chicken SREBP-1 (AY029224) and SREBP-2 amino acid sequences are well conserved in the bHLHZip region (72% identical) and cleavage motifs, whereas they are poorly conserved in other regions. Chicken SREBP1 mRNA is detected in most tissues, and expressed highly in the liver and uropygial gland, both of which have high lipid synthetic activity; also Chicken SREBP2 mRNA is detected in most tissues [19]. In recent study by Yen et al. (2005) identified that the sequence identity of SREBP1 amino acids among Tsaiya duck, chicken, mouse, and human was 90, 76, and 77%, respectively. The sequence identity of SREBP2 between Tsaiya duck, chicken, mouse, and human was 93, 89, and 89%, respectively. The sequence identity of FAS between Tsaiya duck, chicken, goose, mouse, and human was 91, 96, 70, and 71%, respectively. The sequence identity of HMG-CoA reductase between Tsaiya duck and chicken, mouse, and human was 84, 71, and 70%, respectively [34]. So this experiment showed that amino acid sequences of Tsaiya duck genes are very similar to that of chicken, confirming that, genetically, these species are more strongly related than either is to the mammalian species. Furthermore, Tsaiya duck SREBP1 mRNA was expressed in all tissues (adipose tissue, cardiac muscle, skeletal muscle, liver, and ovary) was studied by Yen et al. (2005). They reported that the SREBP2 mRNA concentration was larger in the liver and ovary than in other tissues. The FAS and HMG-CoA reductase mRNA concentrations were high in the liver and low in the other tissues. The duck apoVLDL-II mRNA, as in other avian species, was only expressed in the liver. Thus Liver is the major tissue with high cholesterol biosyntheses activity in avian species [34]. The high hepatic SREBP2 mRNA concentration suggests this gene is involved in upregulation in expression of genes related to cholesterolgenesis, a function similar to that in mammals [35]. Because HMGCoA reductase is a rate-limiting enzyme for cholesterol synthesis, these findings suggest greater cholesterol synthesis in the liver than in other tissues of Tsaiya ducks. In chickens, HMG-CoA reductasemRNAwas detected in most tissues with better concentrations in liver, brain, and ileum than in other tissues [36]. Fatty acid synthase is the key enzyme for de novo fatty acid synthesis. In humans, FAS mRNA is expressed in most tissues, and is highly expressed in the brain, lung, and liver [37]. In pigs, FAS mRNA is expressed in adipose tissue, liver, heart, lung, kidney, and small intestine, and is very expressed in liver and adipose tissue [38,39]. Liver is the major organ for fatty acid synthesis in avian species [40-43]. Therefore tsaiya duck FAS mRNA was highly expressed in the liver and to a lesser extent in other tissues, suggesting that the liver is the major organ of fatty acid synthesis in Tsaiya ducks.

### Effect of lipoporotein- depleted serum (lpds) on the expression and prossing of chick artrial srebps

**Figure 3:**
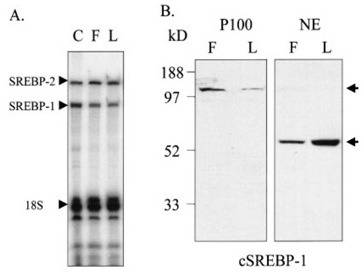
**Effect of LPDS on expression and processing of SREBPs**.

LPDS had no effect on the expression of mRNA coding for either SREBP-2 or SREBP-1 compared with atrial cells from hearts of chicks with 14 days in ovo cultured in FCS. but, Western blot analysis of SREBP-1 in nuclear extracts and membrane preparations of embryonic chick atrial cells cultured in FCS and LPDS demonstrated that growth in LPDS make a marked decrease in the level of a 130-kDa membrane-associated SREBP-1 (Figure 3B, left) and a marked increase in the level of the 60-kDa cleavage product of SREBP-1 in nuclear extracts (Figure 3B, right). These data are constant with the conclusion that LPDS increases SREBP-1 activity via cause on processing of the membrane-associated precursor. It was not possible to study the effect of LPDS on SREBP-2 processing because of poor cross-reactivity with available antibodies [32].

### Choromosomal localization of chicken srebp

In order to search for polymorphisms for SREBP mapping Asef et al. (2003) studied, different introns of SREBP genes were cloned and sequenced including introns 3 (AJ414381), 4 (AJ414382), and 7 (AJ441122) for SREBP-1 and intron 8 (AJ414380) for SREBP-2 that have been submitted to GenBank/EMBL database. They found different introns were located at the same positions as the human ones, and for most of them their lengths were roughly the same between the two species. Chicken vs. human intron lengths are 77 bp vs. 84 bp and 117 bp vs. 85 bp for SREBP-1 introns 3 and 4, respectively, and 689 bp vs. 520 bp for SREBP-2 intron 8. However, intron 7 was found to be divergent in length between chicken and human (641 bp vs. 238 bp) as well as introns 5 and 6 for SREBP-1 and the intron 2 for SREBP-2. The localization of SREBP-1 was determined by segregation analysis in the East Lansing reference family in which the sire was found heterozygous for the 748 bpPCR fragment including intron 7. This localization was confirmed with the 249 bp fragment including intron 4 that was found polymorphic in the family F0/F1/F2 design, already characterized with 27 markers as well as MCW0123. The separation pattern of alleles for intron 4 or intron 7 of the SREBP-1 gene in these different family designs showed that SREBP-1 is to be found on chicken microchromosome 14 at 20 cM from MCW0123 (logarithm of the odds; LOD 8.8). Its human homologue is finding on chromosome 17p11.2 [44]. Chicken chromosome 14 contains two genes (alpha hemoglobin gene and netrin 2 chicken-like) whose human homologues are located on human chromosome 16p13.3. Therefore, the mapping of SREBP-1 to chicken chromosome 14 seems to have revealed a new syntenic block. For SREBP-2, no polymorphism was found between the obtainable cDNA and intronic sequences inside the two chicken reference mapping populations. Considering the localization of human SREBP-2 and the human and chicken map comparison, we assumed that the chicken SREBP-2 must be located on macrochromosome 1 close to N-acetylgalactosaminidase gene. Based on these assumptions, a panel of 84 hybrids was especially selected since their high macrochromosome retention rate. These hybrids were genotyped by PCR amplification of the chicken 898 bp SREBP-2 intron 8 locus, and of seven microsatellite markers located on the chicken macrochromosome1 (LEI0194, MCW0254, MCW0106, ADL0188, ADL0234, LEI068, and MCW0289) [19]. The two-point analysis showed that SREBP-2 is located, as expected, on chicken macrochromosome 1, at 16.3 cR6000 from MCW0289 (LOD = 10). Its human homologue is located on chromosome 22q13 [44]. Chicken chromosome 1 also contains different genes close to SREBP-2, as well as adenylosuccinate lyase and N-acetylgalactosaminidase, whose human homologues are as well located on human chromosome 22q13 thereby showing a good protection of synteny in this region [19].

## Conclusion

The localization of chicken SREBP-1 and SREBP-2 genes will permit their assessment as candidate genes in chicken QTL detection programs focusing on phenotypes related to lipid metabolism. The patterns of SREBP-2 and -1 expression and agreement with motif preservation strongly suggest efficient conservation of the SREBP genes among mammals and chicken. The recognition of the chicken SREBP-2 sequence encoding the mature nuclear form in addition to the chicken SREBP-1 sequence (AY029224) provide significant molecular tools for studying the role of these two transcription factors in the regulation of cholesterol and fatty acid metabolism in avian species.

## Competing interests

The authors declare that they have no competing interests.

## Authors' contributions

All authors have made substantial contributions to this work. FAT designed and drafted the manuscript after literature research. AH supported literature research, drafting and final corrections of the manuscript. All authors read and approved the final manuscript.

## Author's information

FAT is a poultry nutritional and immunological scientist and Ph.D student of Poultry Nutrition Centre of Animal Science, Ferdowsi University of Mashhad, Iran. AH is an associated professor of Poultry Nutrition Centre of Animal Science, Ferdowsi University of Mashhad, Iran.
